# Radial incision and cutting method using a transanal approach for treatment of anastomotic strictures following rectal cancer surgery: a case report

**DOI:** 10.1186/s12957-019-1592-x

**Published:** 2019-03-14

**Authors:** Pramod Nepal, Shinichiro Mori, Yoshiaki Kita, Kan Tanabe, Kenji Baba, Yasuto Uchikado, Hiroshi Kurahara, Takaaki Arigami, Masahiko Sakoda, Kosei Maemura, Shoji Natsugoe

**Affiliations:** Department of Digestive Surgery, Breast and Thyroid Surgery, Graduate School of Medicine, Kagoshima University, Sakuragaoka 8-35-1, Kagoshima, 890-8520 Japan

**Keywords:** Rectal stenosis, Rectal stricture, Anastomosis, Radial incision and cutting, Transanal minimally invasive surgery, Case report

## Abstract

**Background:**

Development of an anastomotic stricture following rectal cancer surgery is not uncommon. Such strictures are usually managed by manual or instrumental dilatation techniques that are often insufficiently effective, as evidenced by the high recurrence rate. Various surgical procedures using minimally invasive approaches have also been reported. One of these procedures, endoscopic radial incision and cutting (RIC), has been extensively reported. However, RIC by transanal minimally invasive surgery (TAMIS) is yet to be reported. We here report a novel application of TAMIS for performing RIC for anastomotic rectal stenosis.

**Case presentation:**

A 67-year-old man had suffered from constipation for 6 years after undergoing low anterior resection for stage II rectal cancer 7 years ago. Colonoscopy showed a 1-cm diameter stricture in the lower rectum. Balloon dilatation was performed many times because of repeated recurrences. Thus, surgical management was considered and the stricture was successfully excised via a RIC method using a TAMIS approach. Postoperatively, the patient had minimal leakage that resolved with conservative treatment.

**Conclusions:**

A RIC method using a TAMIS approach is an effective minimally invasive means of managing anastomotic strictures following rectal cancer surgery.

## Background

Lower rectal surgeries with colorectal or coloanal anastomoses for rectal cancer are complicated by benign anastomotic strictures in up to 22% of patients [1]. Obesity, irradiation, abscess formation, incomplete “doughnut” construction, low-lying anastomosis, use of a stapling device, postoperative leakage, and pelvic infection are reportedly factors related with anastomotic leakage [1, 2]. Digital or instrumental dilatation techniques and endoscopic balloon dilatation (EBD) are currently the preferred treatment methods for such strictures. However, these procedures are sub-optimal because the recurrence rate is high and repeated dilatations may be required [3].

Conventional surgical approaches to treatment are invasive and do not eliminate the risk of recurrence [4]. In 2007, Asada et al. reported radial incision and cutting (RIC), a new technique with promising results [5]. This procedure is feasible, safe, and effective for esophagogastric anastomotic strictures that are refractory to repeated EBD [6]. Here, we discuss a patient with a benign rectal anastomotic stricture following low anterior resection (LAR) that was managed with RIC using a transanal minimally invasive surgery (TAMIS) approach in our university hospital. Although RIC via an endoscopic approach has been reported for treatment of both esophageal and colorectal anastomotic strictures, RIC using a TAMIS approach has not been reported yet. We consider that TAMIS provides a superior operative field, enabling precise incision and adequate excision of such a stricture and consequently better prevents recurrence.

## Case presentation

A 67-year-old man presented with complains of constipation for 6 years. He had undergone LAR for stage II rectal cancer 7 years ago. Postoperatively, he had developed an anastomotic stricture consequent to postoperative leakage and underwent endoscopic balloon dilatation. The symptoms relapsed and after 2 years of first balloon dilatation, it was repeated again. The patient needed the treatment with laxatives for stool softening. Nonetheless, symptoms did not resolve completely and balloon dilatation had to be repeated again with minimal success. Eventually, the patient was referred to our department for surgical management. Then, RIC using TAMIS approach considered the procedure of choice.

**Fig. 1:**
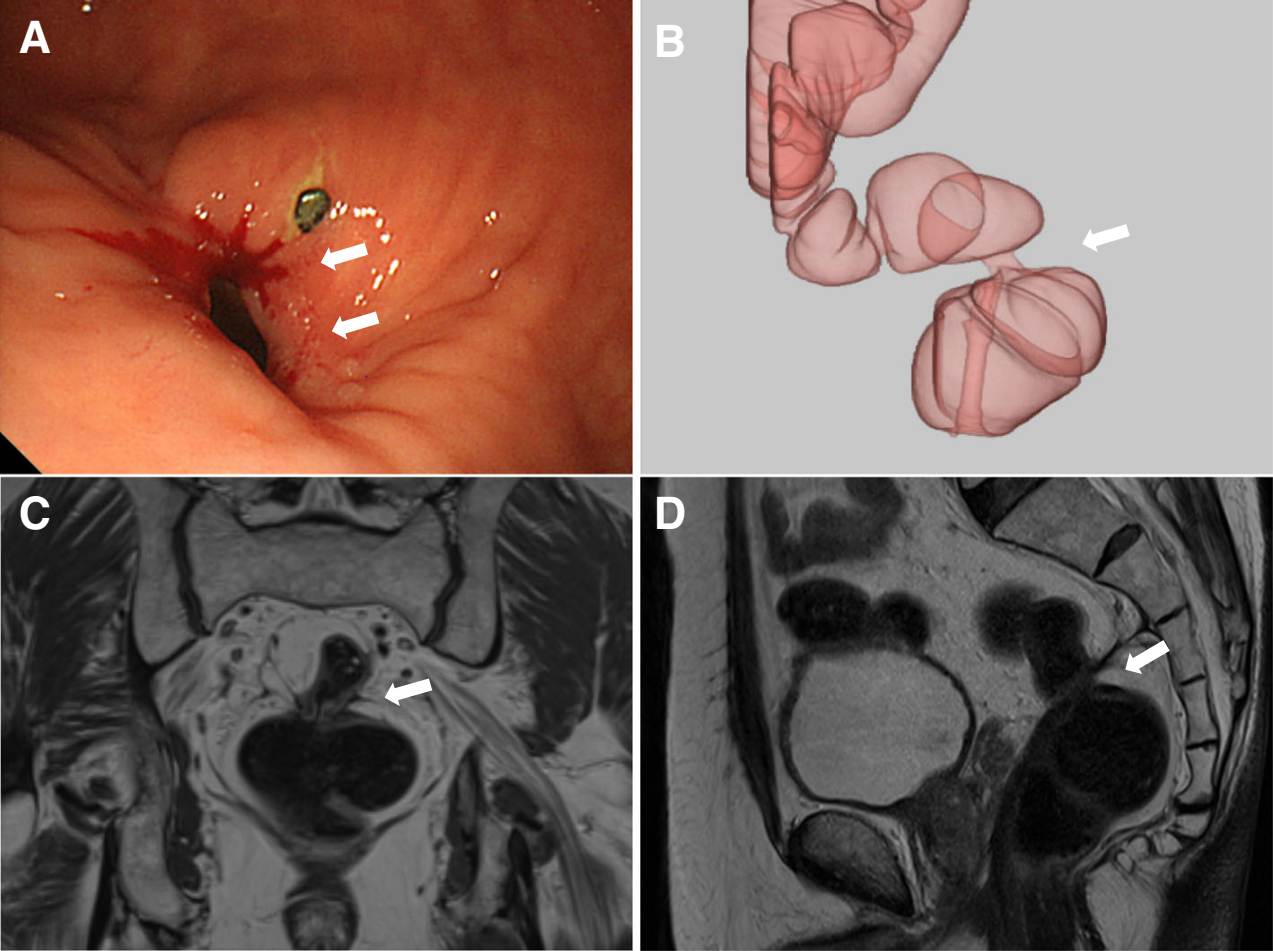
**a** Colonoscopy image showing a stricture in the lower rectum (white arrow). An endoscope of external diameter 9.9 mm could be passed, with resistance, through this stenosis. **b** Preoperative computed tomography–colonography image showing narrowing of the rectum at the stenosis (black arrow). **c**, **d** Magnetic resonance images showing rectal narrowing accompanied by muscular thickening (yellow arrow)

The patient had an unremarkable physical and systemic examination. His BMI was 24.07 kg/m^2^. Medical history revealed hypertension, dyslipidemia, osteoarthritis of the knee, and a past history of pulmonary tuberculosis. The family history was irrelevant. Colonoscopy showed a 1-cm diameter stricture in the lower rectum (Fig. 1a) through which an endoscope with an external diameter of 9.9 mm could be passed with resistance. Preoperative computed tomography–colonography showed narrowing in the lower rectum (Fig. 1b), as did magnetic resonance imaging of the pelvis, which showed rectal narrowing accompanied by muscular thickening (Fig. 1c, d).

**Fig. 2:**
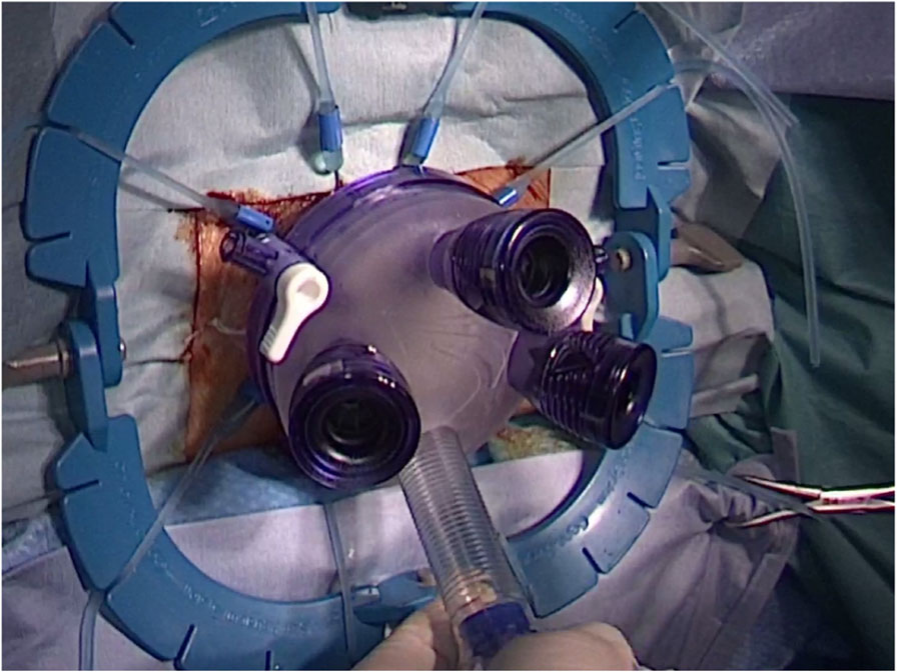
The anal orifice was dilated and a transanal access device was introduced

**Fig. 3:**
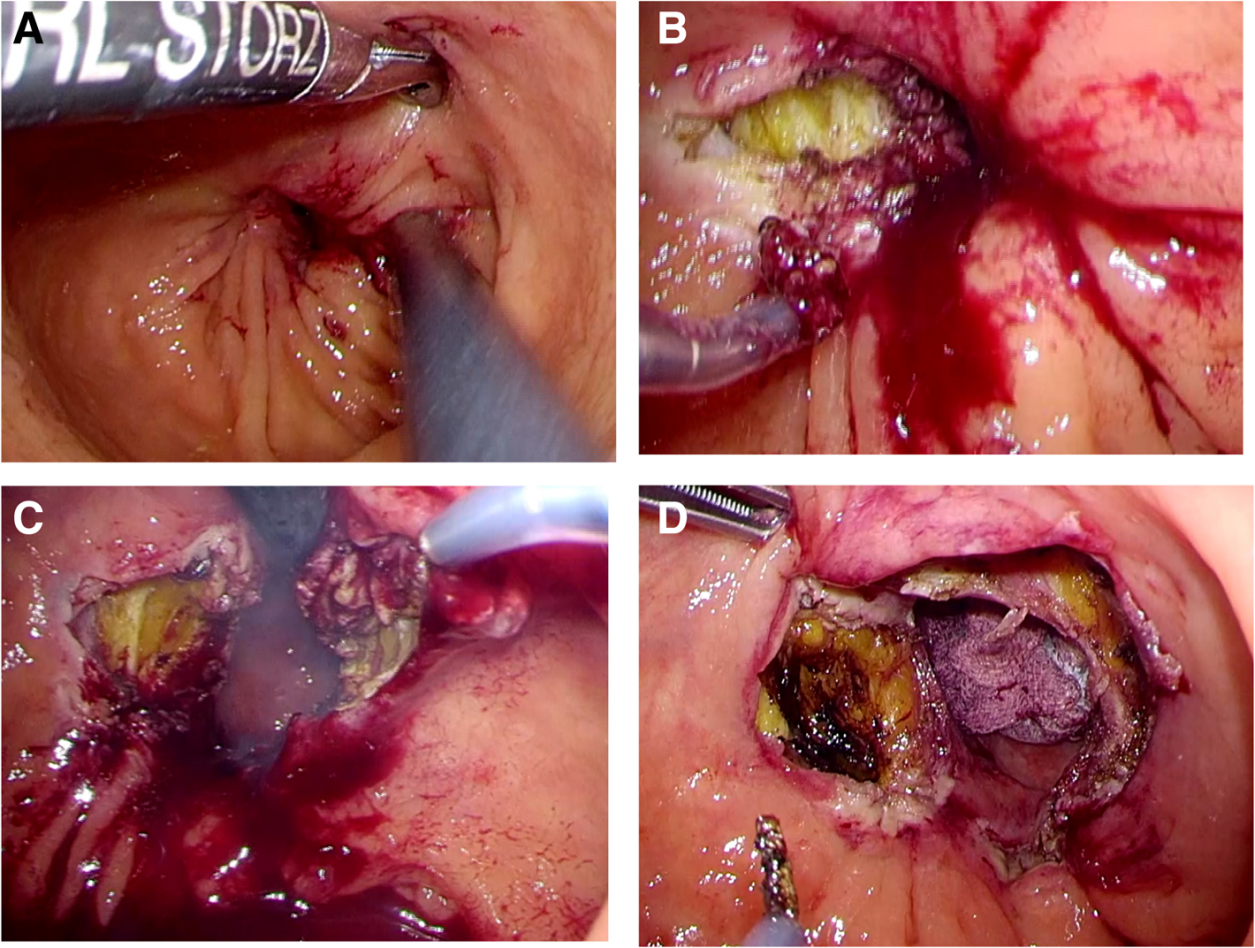
Intraoperative photographs. **a** The stricture has been located in the lower rectum. **b** A full-thickness incision parallel to the axis of the rectum has been made at the 9 o’clock position of the stricture. **c** A similar incision has been made at the 3 o’clock position. **d** The stricture wall was been cut from 9 o’clock to 7 o’clock and 3 o’clock to 6 o’clock

**Fig. 4:**
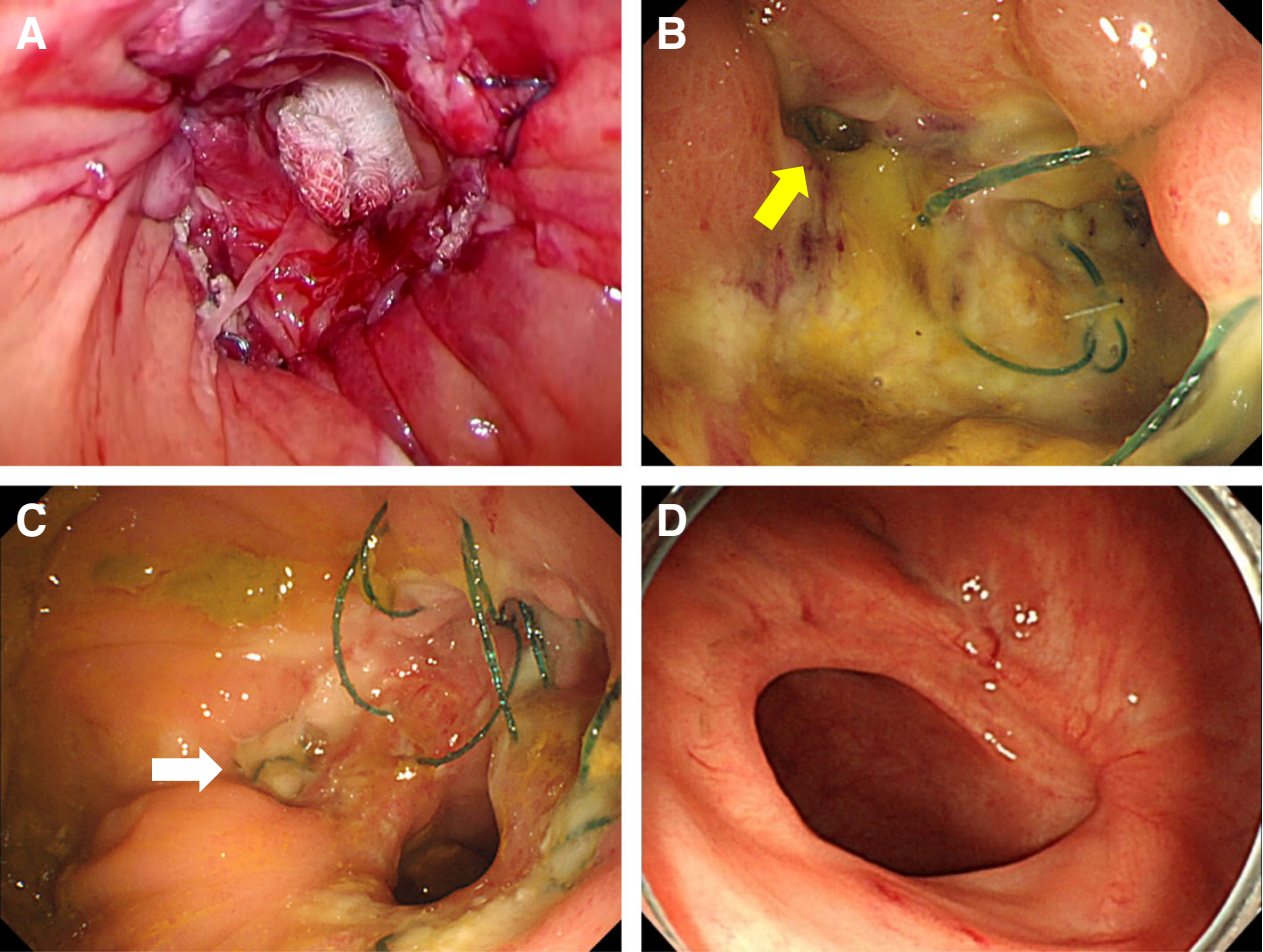
**a** Operative site at the end of surgery. **b** Perforation (yellow arrow) seen in colonoscopy on the 13th post-operative day. **c** The perforation had sealed off when inspected on 26th post-operative day (white arrow). **d** Endoscopic image 1 year after surgery; no stricture is detectable

The patient was placed in a modified lithotomy position and the anus dilated with a self-retaining anal retractor (Lone Star Retractor; Cooper Surgical, Trumbull, CT, USA). A transanal access device (GelPOINT Path; Applied Medical, Rancho Santa Margarita, CA, USA) was introduced (Fig. 2). A pneumorectum was maintained at 12 mmHg with carbon dioxide using an AirSeal platform (AirSeal System; Conmed, Utica, NY, USA), and conventional laparoscopic instruments were used [7]. A 1-cm stricture was located in the lower rectum (Fig. 3a) and full-thickness incisions made parallel to the axis of the rectum at the 9 o’clock (Fig. 3b) and 3 o’clock positions of the stricture (Fig. 3c). The stricture wall was then cut from 9 o’clock to 7 o’clock and 3 o’clock to 6 o’clock (Fig. 3d). After intraluminal lavage with saline, hemostasis was secured. The defect was then closed with 3-0 V-Loc suture (Medtronic, Minneapolis, MN, USA), as shown in Fig. 4a. On the 13th post-operative day, rectal perforation was detected in colonoscopy that resolved with conservative treatment (Fig. 4b). The perforation had sealed off when inspected on 26th post-operative day (Fig. 4c). The patient was discharged on the 33rd post-operative day, 36 days after the admission and attended for regular follow-up without experiencing additional complications. One year after surgery, the patient no longer required medication for constipation, and endoscopic examination showed no stricture (Fig. 4d).

## Discussion and conclusions

Endoscopic balloon dilatation is still the treatment of first choice for benign anastomotic strictures; however, the subsequent recurrence rate is high, often necessitating repeated dilatations [3]. This can lead to severe fibrotic changes in the stricture and ultimate treatment failure [6]. Additionally, there is a high risk of perforation after EBD associated with uncontrolled stretching, inadequate visualization of the entire stricture, and various technical difficulties [8, 9]. Short strictures can often easily be managed with EBD; however, short strictures that are resistant to EBD or larger strictures require surgical treatment [10]. Transabdominal repeat surgery, an ileal pouch advancement procedure, and construction of a permanent stoma are too invasive and should be the last resort. Less invasive options such as stent placement, transanal stricturoplasty, stapler stricturoplasty, resection using transanal endoscopic microsurgery (TEM), and endoscopic transanal resection (ETAR) by urologic resectoscopy are all preferable. An endoscopic electrocautery incision (EECI) technique, in which the stricture is incised radially and the RIC procedure, in which further radial cuts are made in the stricture after incision are additional recently described minimally invasive options [3, 6, 10, 11].

RIC was originally developed for treating esophageal strictures that are resistant to repeated dilatations [5] and was later also used for rectal strictures [12]. The outcomes of RIC are comparable or even superior to those of EECI. According to a review article by Jain et al., the long-term success rate after RIC is 95.8%, whereas that after EECI alone is 95.2%, and after EECI with balloon dilatation 87.8% [8]. The stricture recurrence rate following endoscopic electrocautery with RIC was 0%, following EECI alone was 4.8% and following EECI with balloon dilatation was 12.5% [8]. This review article reports no procedure-related perforation in 186 patients who underwent EECI [8]. Whereas perforation rates of 3.1% and 4.6% following EBD are reported in the study by Park and et al. and Kozarek RA respectively [13, 14]. Thus, EECI techniques have a lower perforation rate than EBD.

We believe that a transanal approach by TEM and TAMIS provides a superior operative field that enables greater precision and more complete resection of a stricture than an endoscopic approach. TEM enables controlled excision of the whole fibrotic ring under full vision, which is cardinal for preventing recurrences [15]. Baatrup et al. experienced no perforations following the excision of rectal strictures by using TEM in six subjects. In one patient, the procedure was converted to balloon dilatation due to very low position of the stricture [15]. However, TEM has not been universally adopted by colorectal surgeons because of the significant cost of the required highly specialized instrumentation and steep learning curve [16]. TAMIS, a hybrid of TEM and single-port laparoscopy, was first reported in 2010 by Atallah et al., who concluded that TAMIS is a feasible alternative to TEM and provides the same benefits at a fraction of the cost [17]. Although the idea of TAMIS is not entirely novel and is based on the similar principle of transanal stricturoplasty of TEM, it is being increasingly adopted, thanks to the requirement for only ordinary laparoscopic instruments and experience with single-port laparoscopic surgery. TAMIS provides minimally invasive access for utilizing the RIC method and ensures precise incisions in a post-rectal surgery anastomotic stricture. It also provides a new strategy for managing recurrent anastomotic stenosis after the use of manual or instrumental dilatation techniques. We were unable to find any reports of using the RIC method with TAMIS approach for benign rectal strictures. Further large prospective studies are needed.

In conclusion, the RIC procedure using a TAMIS approach can be used for minimally invasive management of anastomotic strictures following rectal cancer surgery and is a promising means of managing recurrent stenosis.

## Abbreviations

EBD: Endoscopic balloon dilatation
EECI: Endoscopic electrocautery incision
ETAR: Endoscopic transanal resection
LAR: Low anterior resection
RIC: Radial incision and cutting
TAMIS: Transanal minimally invasive surgery
TEM: Transanal endoscopic microsurgery
